# Building a Comprehensive Sickle Cell Disease Program in Western Kenya: A Decade of Experience and Growth

**DOI:** 10.5334/aogh.4725

**Published:** 2026-01-20

**Authors:** Festus Njuguna, Carole Kilach, Cyrus Njuguna, Erick Ayaye, Christopher Wanjiku, Rachael Korir, Consolata Bor, Nancy Midiwo, Everlyne Aliwa, Elvis Oburah, Samuel Mbunya, Joseph Kipkoech, Mary Ann Etling, Tyler Severance, Charles Nathaniel Nessle, Terry Vik, Manjusha Kumar, Chris Roberson, Anne Greist

**Affiliations:** 1 Academic Model Providing Access to Healthcare, Eldoret, Kenya; 2 Moi University School of Medicine, Department of Child Health, Eldoret, Kenya; 3 University of North Carolina, USA; 4 Indiana Hemophilia and Thrombosis Center, USA; 5 University of Missouri, School of Medicine, USA; 6 University of Michigan, Department of Pediatrics, Division of Hematology/Oncology, Ann Arbor, MI, USA; 7 Fogarty International Center, National Institute of Health, Bethesda, MD, USA; 8 Indiana University, USA

**Keywords:** Sickle cell disease, comprehensive care, low resource settings

## Abstract

*Background:* Globally, approximately 515,000 infants with Sickle Cell Disease (SCD) are born every year. Approximately 80% of these cases occur in Sub-Saharan Africa (SSA) annually, including 14,000 newborns in Kenya. In SSA, 50%–80% of children will die before the age of 5 years due to a lack of comprehensive SCD care compared to 3% in better-resourced settings.

The Academic Model Providing Access to Healthcare (AMPATH) SCD Program started in 2010 as a partnership between Moi University, Moi Teaching and Referral Hospital (MTRH), and Indiana Hemophilia and Thrombosis Center (IHTC) with a goal to improve access to comprehensive SCD care by increasing capacity through training, clinical care, research, and advocacy.

*Findings:* The program has trained over 5,000 healthcare workers on different aspects of SCD through face-to-face instruction, virtual training and one-on-one mentorship programs. Early infant screening and support for access to medications like hydroxyurea and antibiotics have been key in improving clinical care. The program has also participated in several research projects and has been a strong advocate for the provision of comprehensive SCD care by the health facilities within the high SCD burden areas in Kenya and the Ministry of Health.

*Conclusion:* The strategies implemented by the program can serve as a template for establishment of SCD care programs in similar resource-limited settings.

## Introduction

### Sickle cell disease background

Sickle Cell Disease (SCD) is an inherited genetic disorder of red blood cells, resulting in abnormal sickle hemoglobin expression, which causes a range of health problems, decreased life expectancy, and a high mortality rate. Globally, an estimated 515,000 children are born with SCD every year, with 405,000 cases occurring in Sub-Saharan Africa (SSA). The number of children born with SCD increased by 27% between 2000 and 2021 in SSA. The global prevalence of SCD in 2021 was estimated at 7.74 million cases, including 5.7 million cases in SSA. In 2021, there were 376,000 deaths among people living with SCD, of which 265,000 (70%) occurred in SSA. Notably, SCD was the 11th leading cause of death among children under 5 years old in this region [1].

Throughout Kenya, an estimated 14,000 infants with SCD are born annually [2]. Counties near the lake and coastal regions are hypothesized to have a disproportionately high SCD prevalence due to malaria endemicity [1]. In addition, the true disease burden of SCD in Kenya, including both prevalence and mortality, is underestimated due to the absence of a national newborn screening program.

The high mortality rate in individuals with SCD in SSA is multifactorial. Many children remain undiagnosed in early childhood due to lack of reliable screening programs, and death is often due to preventable or treatable infections, including malaria and bacterial sepsis. Limited access to care and lack of comprehensive SCD management programs also contribute to preventable death. Failure to detect known disease complications and decreased hydroxyurea utilization are two potential consequences of a lack of accessible care [3].

The WHO resolutions for individuals with SCD adopted in 2006 and 2010 committed to increased awareness, promotion of equitable access to providers, provision of technical support for prevention and management, and support of research to improve quality-of-life for affected individuals [4]. In response, there are several examples of international collaborations to address disparities in SCD by increasing utilization of newborn screening and access to care. Mortality from SCD decreases with early diagnosis, and outcomes improve when countries invest in implementing comprehensive SCD programs [5]. Local efforts to include community health workers aim to improve psychosocial support for individuals with SCD as well [6–9].

Despite the innovative adaptations to improve care delivery and diagnosis of individuals with SCD, disparities in equitable care remain in SSA. While descriptions of a single facet of a program have previously been published, to our knowledge, the detailed description of the evolution of a comprehensive SCD program does not exist. This article describes the effort to train healthcare workers, improve clinical care, augment research efforts, and increase advocacy through a decade of experience to establish a comprehensive SCD program in Western Kenya via an international collaborative effort. The institutional ethics committee granted approval waiver for the writing of the article.

### Program description

**Figure 1:**
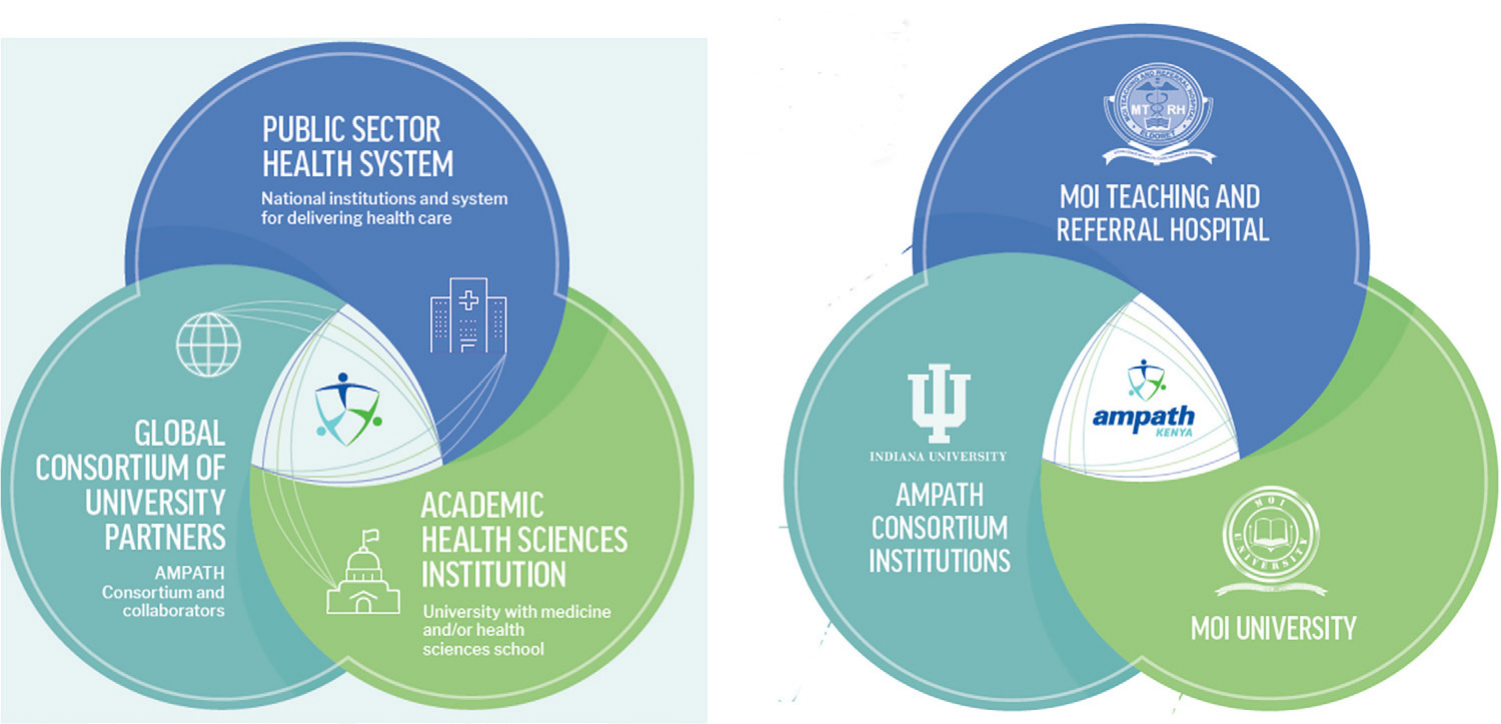
AMPATH Model.

The Academic Model Providing Access to Healthcare (AMPATH) consortium is an international partnership between Moi University, Moi Teaching and Referral Hospital (MTRH), and academic institutions globally led by Indiana University [10]. The consortium focuses on improving the health of people in underserved communities by working in partnership with academic health centers, ministries of health, and others to build public sector health systems and promote well-being (Figure 1).

Guided by the principle of leading with care, it achieves this through delivering and sustaining effective healthcare service, reducing health disparities and addressing social determinants of health, developing and strengthening human capacity through training and education, advancing research that improves health, and strengthening partner institutions.

**Figure 2:**
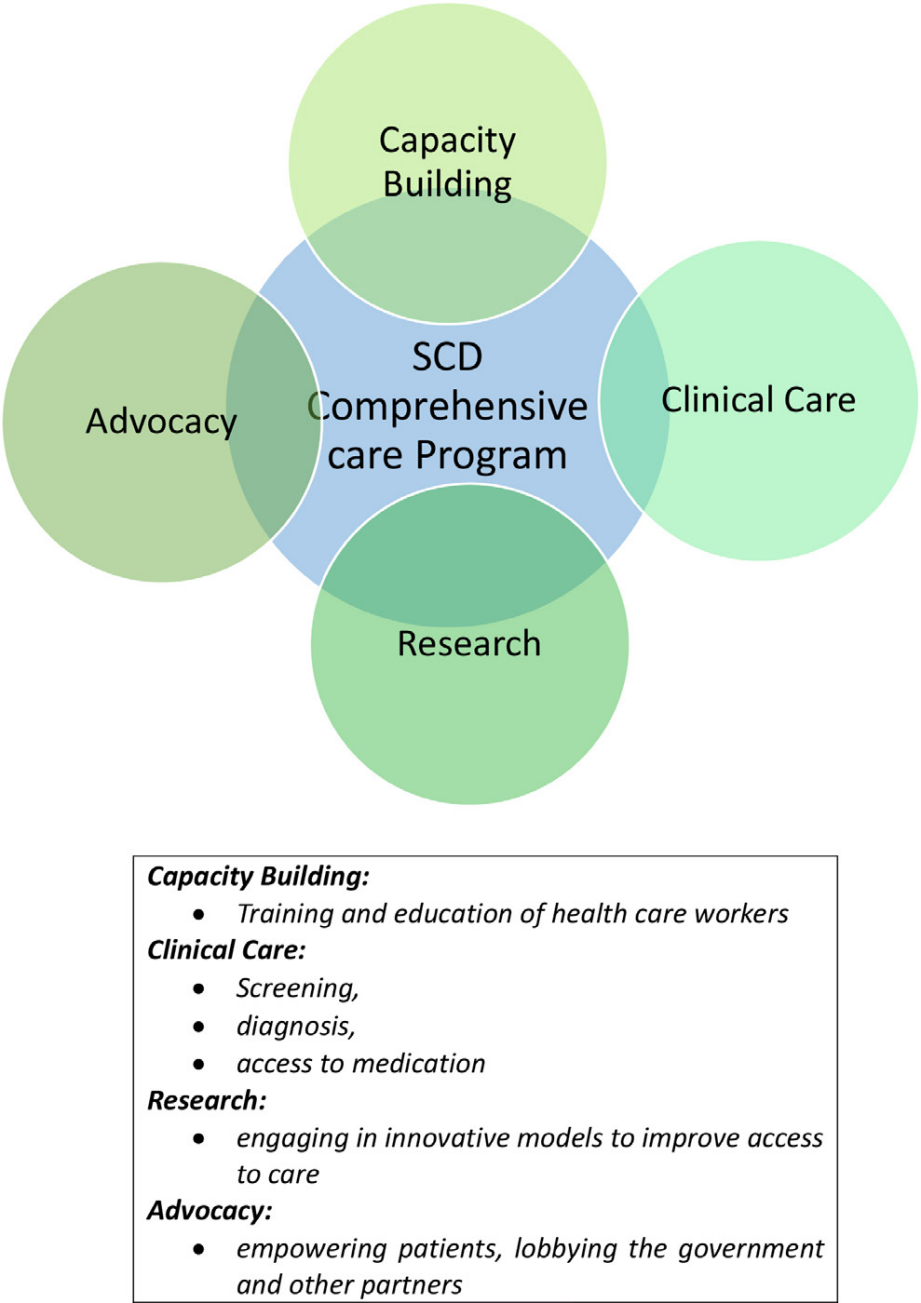
AMPATH SCD program: Thematic areas.

The AMPATH Hematology Program was established in 2010 with the goal of improving the lives of people living with hematologic conditions including SCD by improving access to comprehensive care through capacity building, collaborative networks, advocacy, and innovation. The main objectives of the program are to increase the number of healthcare workers and patients with knowledge on SCD in Western Kenya, increase the number of people accessing SCD care in Western Kenya, and increase research capacity through training and implementation (Figure 2).

**Figure 3:**
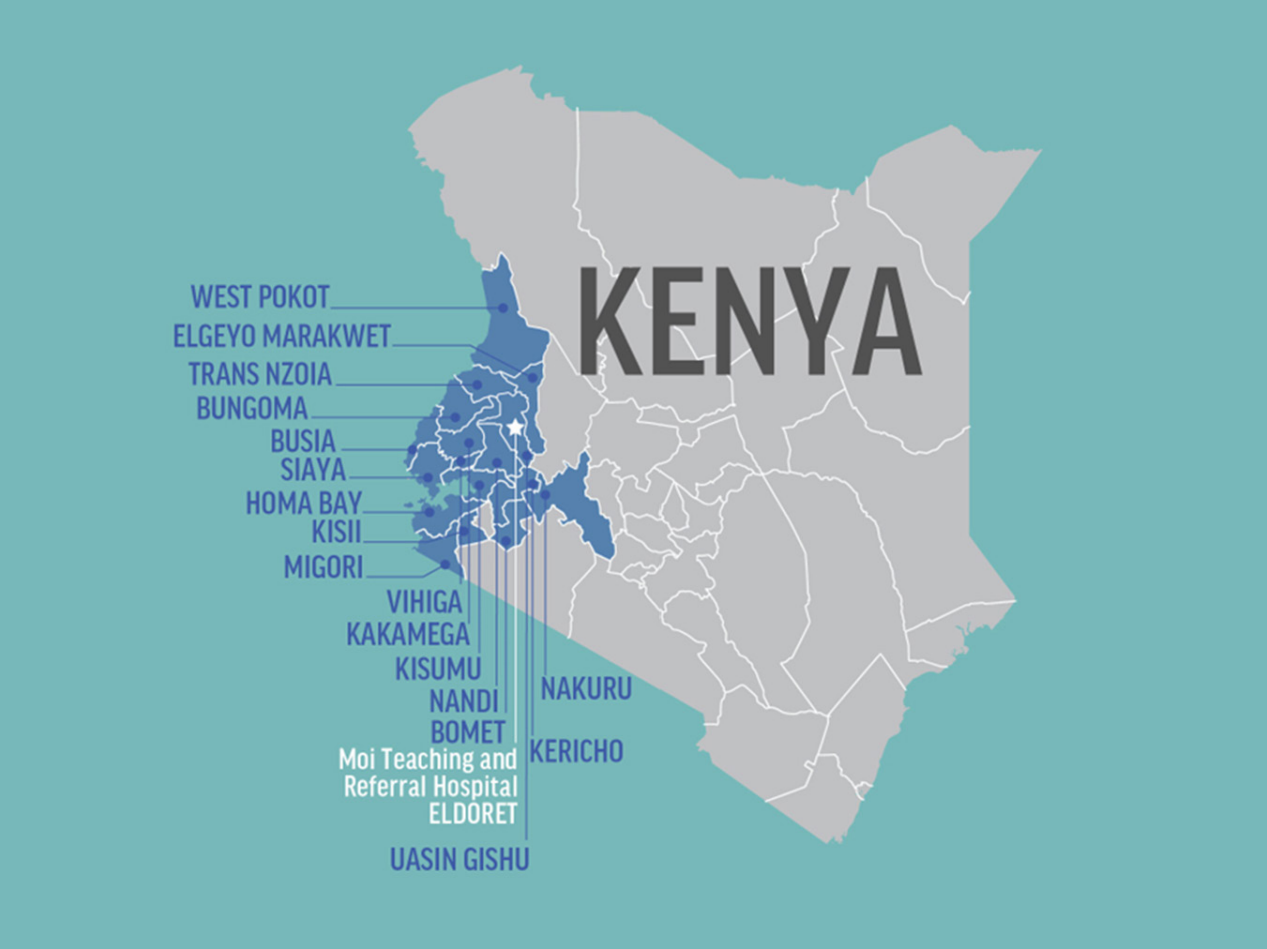
AMPATH catchment area.

The program is based in MTRH in Eldoret, Kenya and supported by the Indiana Hemophilia and Thrombosis Center (IHTC) and AMPATH partners. The AMPATH Hematology Program has a multidisciplinary team of physicians, nurses, physical therapists, psychological counselors, pharmacists, child life specialists, and healthcare administrators. The AMPATH program focuses its efforts within Western Kenya (Figure 3) and serves a catchment area of over 24 million people. The region includes counties that are within the Lake Victoria Basin, a malaria endemic area leading with a high SCD burden locally [10].

## Program Implementation

### Training and capacity building

**Chart 1:**
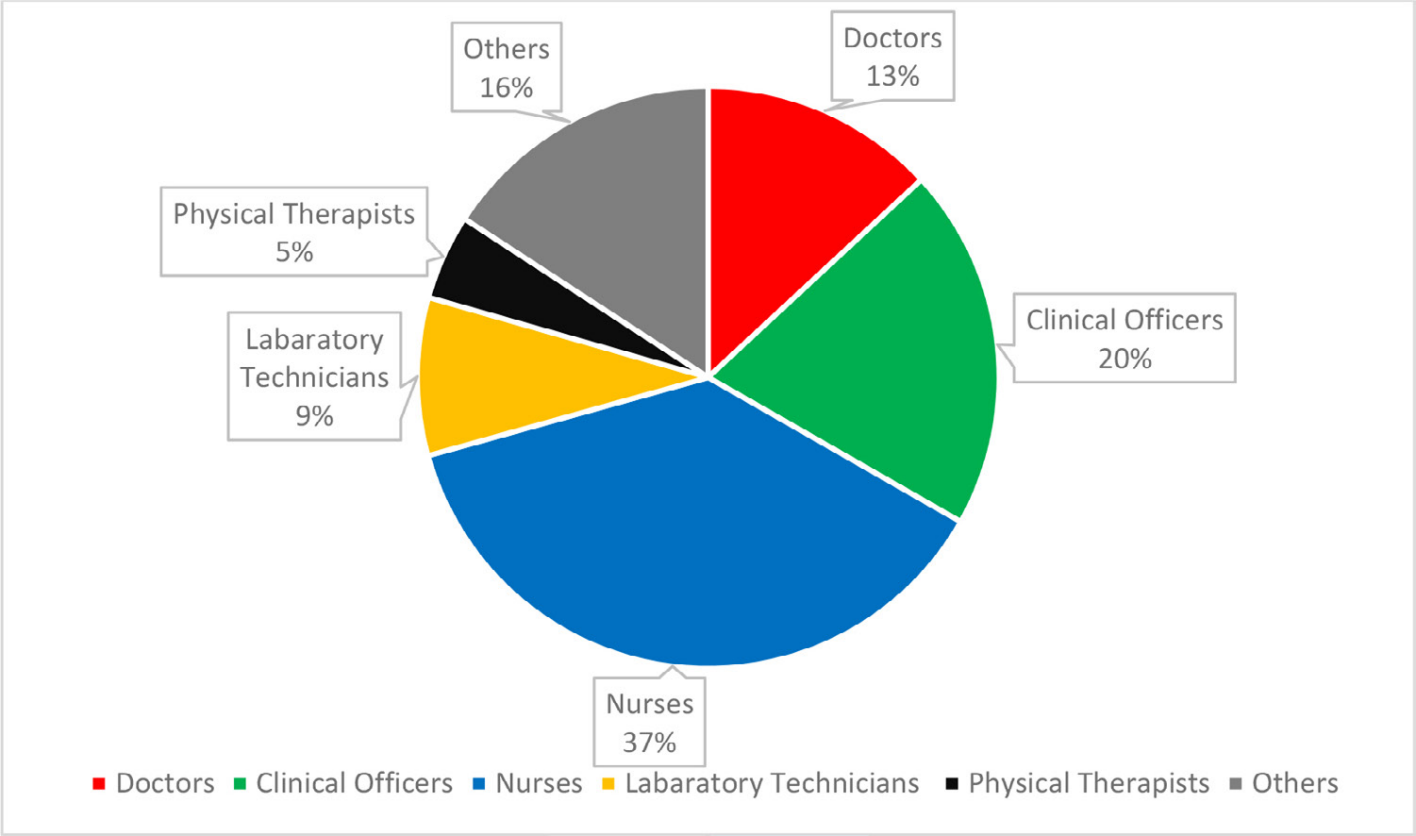
Cadres of healthcare workers trained on SCD from 2015 to 2023 (N = 4022).

Following its inception in 2010, the program provided training to healthcare workers of all cadres, including doctors, clinical officers, nurses, pharmacists, and laboratory technicians, as represented in Pie Chart 1. In-person training courses were conducted by a team of experts from local institutions as well as the international partners. The program adopted and developed a curriculum that evolved and responded to the needs of stakeholders. The primary themes covered in the training were screening, diagnosis, epidemiology, genetics, clinical presentation, management of acute and chronic complications, and comprehensive care of SCD (Appendix 1). Initial regions targeted for training emphasized geographic proximity to MTRH and counties with high prevalence of SCD. A leader at each targeted healthcare facility was approached with a request to support a local training session. The leader identified approximately 50 attendees from diverse professional cadres and sub-county clinics. Each training was delivered over a one-day period with a pre-test and a post-test used to evaluate knowledge acquisition. The participants received a copy of the presentations for future reference. They also received certificates of attendance that they could use for Continuous Professional Development credits toward annual license renewal. Although the training programs were well received, notable limitations include a lack of follow-up and difficulty measuring clinical impact.

In addition to the outreach efforts, the team developed in-person training sessions for selected health care providers to travel to MTRH for a 2-week mentorship program. Like the outreach visits, the mentorship focused on providing knowledge and skills to build a multidisciplinary SCD clinic. This ultimately led to extended visits by entire clinical teams from neighboring hospitals emphasizing the multidisciplinary approach of a SCD program. So far, we have hosted teams from three facilities, which included at least a clinician, a nurse, and a physiotherapist.

With the COVID-19 pandemic in 2020, the training sessions transitioned to a virtual platform for continuity, leading to greater participation by learners and stakeholders. Project ECHO (Extension for Community Healthcare Outcomes) for SCD was launched in June 2022 as a monthly virtual educational platform to provide in-depth SCD skills and knowledge for all cadres of healthcare workers [11]. Topics varying from screening, management of acute and chronic complications are covered in the different sessions as highlighted in Appendix 2. The ECHO program utilizes a hub-and-spoke educational model, consisting of a multidisciplinary expert hub team engaging with spoke sites. Targeted learners include healthcare providers and other stakeholders in medically underserved communities. Held monthly, each ECHO session is 60–90 minutes in length and includes a didactic lecture followed by 1–2 deidentified interactive case presentations submitted by spoke sites. The topics of discussion have included antibiotic prophylaxis and the role of vaccinations in SCD, hydroxyurea use, acute pain crisis management, and treatment of fever and infection. The Kenya SCD ECHO has addressed the need for a continuous, sustainable, accessible training platform with a mechanism for evaluation of impact and effectiveness.

In total, these education sessions have reached over 5,000 healthcare workers throughout Kenya supporting continued expansion of educational initiatives [11].

### Clinical care

Improvements in care offered to individuals with SCD have led to great improvement in life expectancy in high-income settings [12]. By extension, clinical care has been a core component of the comprehensive SCD program at MTRH since its inception. The AMPATH program initially set up a comprehensive SCD clinic at MTRH, with subsequent support extending to other clinics within the AMPATH network (Figure 3). The clinical support provided by the AMPATH Hematology Program team varied based on needs of the network clinics. This included logistical and educational materials, site visits and mentorship, and increased access to necessary SCD medications such as hydroxyurea.

**Graph 1:**
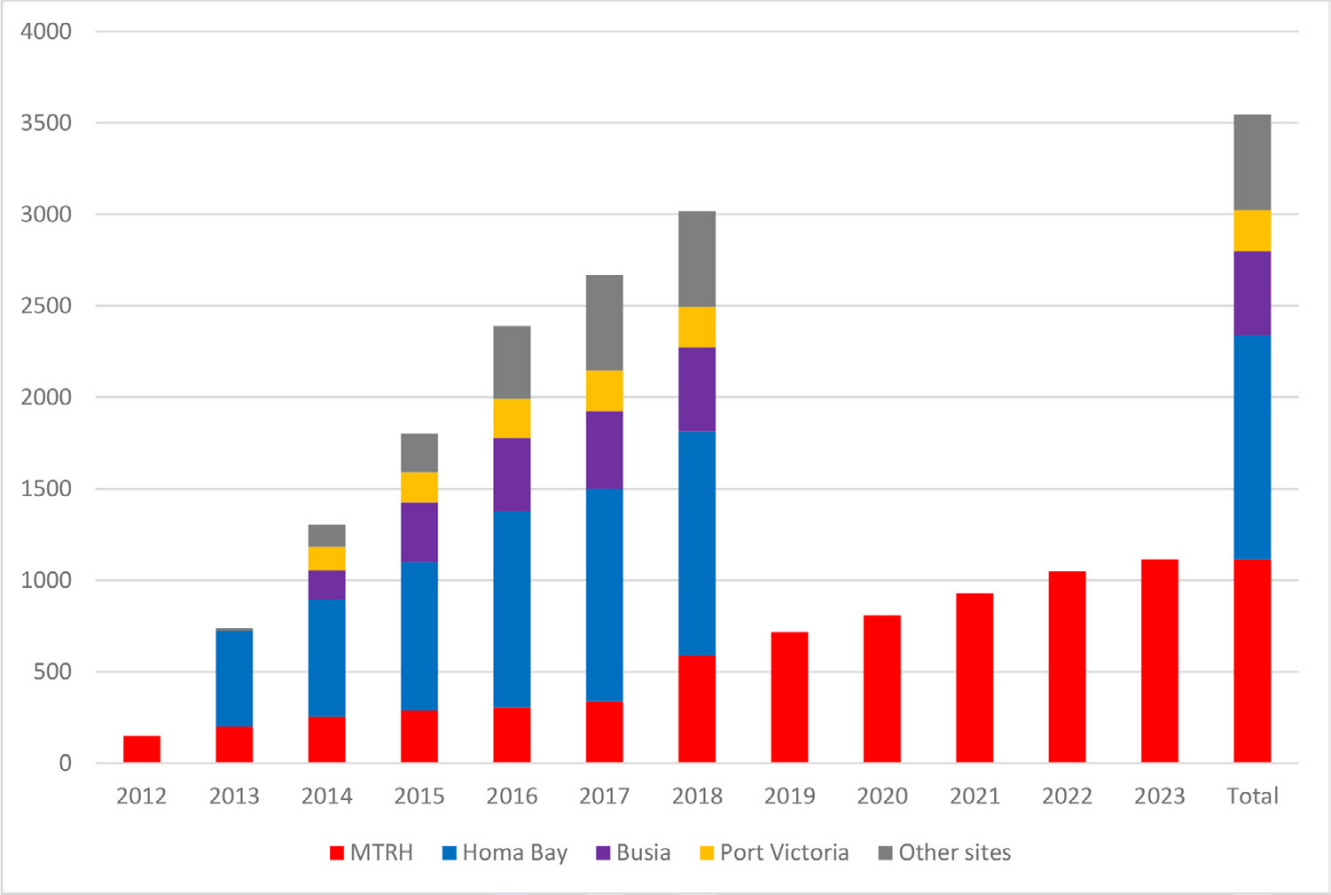
SCD patients enrolled to care in different clinics from 2012 to 2023 (Total of 3545).

These efforts led to an increase in the number of patients with SCD on follow-up care with over 3,500 patients linked to care from 2012, as shown in Graph 1.

Recognizing the criticality of universal newborn screening (NBS) [1], our team piloted several NBS programs. In the initial phase, isoelectric focusing was used to screen over 11,000 newborns at MTRH where 0.8% of the newborns were diagnosed with SCD. We also did pilot screening programs at other sites in Western Kenya (Kisumu and Homa Bay County Hospitals) using isoelectric focusing. Although implemented successfully, the main challenge was connecting those with a positive screen into care due to delayed result reporting after specimen collection and difficulties connecting patients to care.

Eventually, point of care (POC) tests for SCD became available for use by the program. POC tests provide a diagnosis in real time and allow for immediate counseling by team members, leading to better integration into care. The high sensitivity and specificity that has been demonstrated by POC tests makes them very applicable to screening for SCD in resource-limited settings [13]. To capture children born outside of medical facilities, we also targeted early childhood immunization clinics to offer additional POC testing.

**Graph 2:**
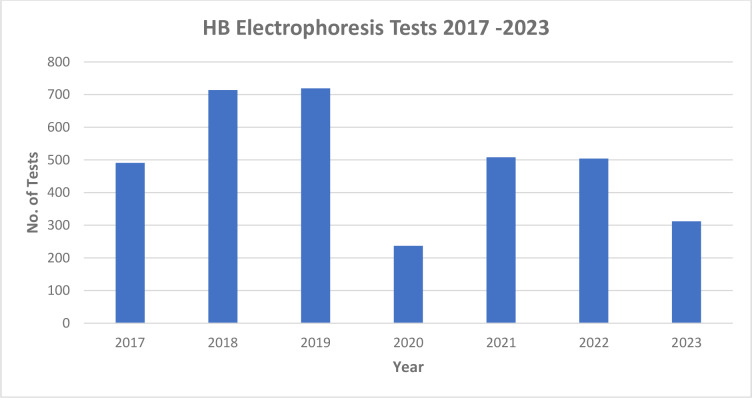
Hemoglobin electrophoresis tests done from 2017 to 2023.

For diagnostic confirmation, the program collaborated to procure equipment for hemoglobin electrophoresis. We established a system for shipping samples using a courier company, relaying results back to the referring facilities via email. This increased access to confirmatory testing without requiring patients to travel to MTRH. Initially, electrophoresis was provided without charge to patients with suspected SCD or a positive POC test. Ultimately, this was not sustainable, and in 2018 a subsidized fee of $7.50 USD was introduced. However, this fee remained substantially lower than the average laboratory cost in the region. With increased use of POC tests the utilization of electrophoresis decreased over time as shown in Graph 2. From 2015 to 2021, we did over 4,000 hemoglobin electrophoresis tests of which 69.2% confirmed SCD (SS), 20.2% revealed sickle cell trait (AS), while 10.6% were normal (AA).

Provision of medication, especially hydroxyurea, is critical for reducing the frequency of acute and chronic complications of SCD. Moreover, infection prevention through the use of penicillin prophylaxis and vaccination reduces mortality in persons living with SCD [13]. There was minimal use of hydroxyurea in Kenya prior to 2010 due to limited availability and excessive cost with an average price of $0.50 per 500 mg capsule. A study performed in a district hospital in Kenya spanning 2003–2010 reported no use of hydroxyurea [14]. The AMPATH Hematology Program began by sourcing hydroxyurea using funds from an international collaborator. Due to limited supply, patients were selected based on restrictive criteria (frequent painful crises, stroke, acute chest syndrome, or frequent transfusions). Over time, the supply increased, allowing expansion of eligibility to include all children with SCD older than 9 months as per the updated treatment standards [15].

To improve sustainability, the program introduced a fee of roughly $4 per family per month as a copay for medication. A care medication package was subsequently created, and this fee also catered for penicillin, folate, and proguanil where indicated, using a revolving fund pharmacy (RFP) model [16]. Currently, there are 18 RFPs across Western Kenya in Uasin Gishu, Busia, Homa Bay, Bungoma, Trans Nzoia, and Kisumu counties that are able to provide these medications. Efforts were made through partnerships with pharmaceutical companies to procure a reliable supply of hydroxyurea at the lowest wholesale price, approximately $0.15 per 500 mg capsule. Availability of pneumococcal and meningococcal vaccines, at no or subsidized cost, also improved through support from international collaborators. Over a period of 10 years, the program has been able to procure over 1,477,000 capsules of hydroxyurea; 500,000 tablets of penicillin; and 700 vials of vaccines.

### Research

Using the clinical care infrastructure, the program has also prioritized SCD research and dissemination of results. We participated in a study to validate a POC testing kit for SCD at a large outreach site. Seven hundred children under 5 years were enrolled, and the results were presented at the American Society of Hematology Annual Scientific Meeting [17]. The POC screening sensitivity was 92.1% with a specificity of 95.0%. Using the same POC, we screened children under the age of 5 years in one of the outreach clinics where 93.4% of those who screened positive were initiated on care. The program also conducted an investigator-initiated clinical trial evaluating the effectiveness of daily proguanil (the standard of care), monthly sulfadoxine/pyrimethamine plus amodiaquine, or monthly dihydroartemisinin-piperaquine as malaria prophylaxis in individuals with SCD [18].

#### Advocacy and policy

To improve care across the region, it is equally important to empower patients and caregivers [19, 20]. The program developed educational materials focused on the genetics of SCD, signs and symptoms of the disease, management of complications, and the importance of medication and clinic adherence. Community education in the region has helped in destigmatizing the disease.

A key pillar in advocacy has been the creation of support groups comprising patients and caregivers at facility, county, and national levels. The program contributed technical expertise to the creation of a national umbrella patient organization (Sickle Cell Federation of Kenya). The advocacy groups educate and provide a platform to discuss issues around patient care, caregiver challenges, and psychosocial issues. Each group conducted meetings independently and incorporated asynchronous communication forums (such as WhatsApp) to provide increasing accessibility and continuous engagement.

In 2018, a greater effort was made to include healthcare workers in county and national advocacy. This effort, which also featured patients, caregivers, and other healthcare leaders, brought together stakeholders to align government leaders toward affordable, accessible, and sustainable SCD care through centers of excellence. The advocacy efforts have also been carried out through schools (providing the school educators and students with information regarding SCD), faith-based organizations, and local leaders, further helping to address stigma issues associated with SCD in the community. Finally, the AMPATH program has participated with SCD support groups regionally and nationally to raise awareness and advocacy through the World SCD Day celebrations, media engagement through radio and television, awareness walks across the towns, and social media campaigns.

## Lessons Learned

Spanning the past decade, the program has successfully built the infrastructure and key components of a comprehensive SCD clinic and regional network. However, there were several valuable lessons during the implementation of the program.

### Collaboration/Partnership

The program was founded on a strong partnership as per the AMPATH model. The international partners (mainly IHTC) provided the initial support and built the capacity of the team at MTRH and Moi University, which has enabled them to support the expansion of the program. The engagement of the local and the North American collaborators was as equal partners. The teams collectively identified the local needs and discussed the best ways to have appropriate and sustainable solutions. The collaboration involved various departments and cadres, including clinical, nursing, laboratory, pharmacy, physiotherapy, child life, and counselors, with creation of leads in these sections.

Over the years, the engagements with international and local partners and stakeholders have led to notable outcomes including the establishment of a comprehensive SCD care model in the country, firmly embedded within the national healthcare policy framework. Over the years, the national government implemented the national SCD management guidelines and has developed an SCD Infant Screening Policy. At county level, there are local government-led SCD care programs in selected counties.

**Table:** 

	CHALLENGES/LIMITATIONS	MITIGATION
**Training and Capacity Building**	Lack of sufficient education, training, and capacity to care for people with SCD Limitations of the in-person conference style model, the long-term learning and engagement was ultimately limited, and without continuous learning opportunities, much of the knowledge gained was lost	Training and stakeholder engagement to increase the level of commitment Identifying local facilitators/champions at various hospitals/clinics to continue with local training/education Regular virtual training and case-based discussions using the ECHO platform
**Clinical Care**	Sites relied heavily on the program for medications and diagnostic supplies Unavailability of reasonably priced medications Low overall demand for Hydroxyurea in many SSA countries Expensive laboratory tests for confirmation of diagnosis	Establishment of revolving fund pharmacies Lobbying the drug distributors to reduce cost of medication Lobbying the government for inclusion of SCD medication into an SCD care package under the national public health insurance scheme Utilization of courier services to transport samples where confirmatory hemoglobin electrophoresis testing was required Use of point of care testing
**Research**	Inability to conduct/participate in research and publish	Training the staff in research/writing skills Having dedicated time to conduct research Writing grants to provide staff who can focus on research
**Advocacy**	Lack of technical skills by parents/caregivers on advocacy Low prioritization of SCD by government	Training of persons living with SCD/caregivers on advocacy Identification and engagement of key government officials and other stakeholders on SCD

## Way Forward and Sustainability

Looking into the future, improved SCD care throughout the country remains the goal of the program.

Building on the four pillars of the program, capacity building, care, research, and advocacy, sustainability is becoming an essential part of our strategy going forward. The program has initiated several efforts to support a life beyond its current mandate, which focuses on improving access to diagnostics and essential medicines, advocating for policy changes, and empowering patients and caregivers.

1. **Collaboration**
  - With the success of collaborative efforts, the program is keen to build on the established partners and continue to engage with the government and institutions/facilities and other partners to ensure SCD care; early diagnosis and comprehensive care is part of routine care.
2. **Training and capacity building**
  - Continued integration of in-person and virtual trainings.
  - Adapting the training of trainer’s model to increase the number reached.
  - Continued identification and utilization of local champions at both national and community level.
  - Advocating for retention of the trained staff to continue working in the SCD clinical areas.
  - Focusing on building the capacity of the community health volunteers who provide a vital link between the community and the healthcare system.
  - Lobby for SCD content to be incorporated in the training curricula of various medical training institutions.
3. **Clinical care**
  - Support the government in the expansion of newborn screening by providing technical support based on the knowledge and skills gained from the pilot SCD screening studies.
  - Support the clinical personnel in various facilities with decision-making through telephone consultations and creation of online groups for healthcare workers, e.g. WhatsApp group.
  - Engage the public health insurance scheme to provide coverage of SCD care.
  - Continue to engage pharmaceutical partners to increase the access, availability, and affordability of SCD commodities in health facilities.
  - Expand and strengthen the revolving fund pharmacies.
4. **Research**
  - Training of staff on research and grant writing.
  - Have clear research questions and data collection tools.
  - Apply for research grants and also allow staff to have dedicated time for research as part of their duties.
5. **Advocacy**
  - Continue to support the capacity building of SCD community to advocate.
  - Identify and engage SCD champions at different levels: international, national, and local.
  - Support the National SCD Federation.

## Conclusion

After more than a decade of collaboration, engagement, and research, we have established a comprehensive SCD clinic in MTRH with a network of clinics throughout Western Kenya. The AMPATH Hematology Program pillars of education and training, care and collaboration, and research and advocacy have effectively empowered local communities to continue improving care for individuals with SCD in Western Kenya. This model can serve as a replicable pathway to disseminate across SSA and in other resource-limited settings around the world.

## Additional File

The additional file for this article can be found as follows:

Supplementary material. Supplementary appendix.

This finding of multiple local introgression events has at least three key implications.
