# Supplementary material for: Building a Comprehensive Sickle Cell Disease Program in Western Kenya: A Decade of Experience and Growth
Source: Ann Glob Health. 2026 Jan 20;92(1):7. doi: 10.5334/aogh.4725 (PMC12829446; doi:10.5334/aogh.4725)
Supplement: Supplementary material. — Supplementary appendix. [file agh-92-1-4725-s1.pdf]

Appendix

Appendix 1- In person SCD training schedule in 2022

Sickle Cell Disease Training for Healthcare Workers

April 13th 2022.

WEBUYE
Venue: Minata Hotel

Time	Topic	Presenter
8:00 - 8:30am	Registration, Welcome, Introduction and Expectations	Cyrus
8:30 – 9:00am	Pre -test	Cyrus
9:00 - 9:30am	Introduction, Epidemiology, Genetics Pathophysiology of SCD	Dr. Millicent Wanyama
9:30-10:30am	Diagnosis - Clinical Features Screening & Laboratory Diagnosis of SCD	Dr. Carole Kilach
10.30am-11:00am	**Tea break**	
11:00-12:00	Management of Acute Complications in SCD	Dr. Beatrice Melly
12:00 – 12:30pm	Health Maintenance in SCD	Prof Anne Greist
12:30 - 1:00 pm	Patient Education in SCD	Jeanne Saggar
**1:00 - 2:00pm**	**Lunch**	
2:00 – 3:00pm	Management of Chronic Complications in SCD	Dr. Anthony Betbadal
3:00 – 3:30pm	SCD Service Delivery: Important aspects to have in place	Cyrus Njuguna
3:30 – 4:00pm	Post Test	Cyrus
**4:00**	**Tea and Departure**	

Appendix 2: SCD project ECHO Schedule 2022-2023

Y1 SCD ECHO 2022-2023 Programme

Quarter	Date	Session Title	Speaker	Participants
2	21/6/2022	Sickle Cell Disease and Telehealth	Dr. Tyler Severance- Univ Missouri	70
3	19/7/2022	Antibiotic prophylaxis and role of vaccinations in SCD	Dr. Festus Njuguna-Moi Univ	75
3	23/8/2022	Hydroxyurea use in SCD	Dr. Carole Kilach- AMPATH	67
3	20/9/2022	Acute pain crisis management in SCD	Dr. Gilbert Olbara- MTRH	66
4	18/10/2022	Management of fever/infection in SCD	Dr. Dickens Lubanga-Bungoma	61
4	22/11/2022	Management of acute anaemia and role of blood transfusions in SCD	Dr. Millicent Wanyama-Webuye	45
4	20/12/2022	SCD Laboratory diagnosis	Racheal Korir- AMPATH	60
1	24/1/2024	Stroke in SCD	Dr. Manjusha Kumar-IHTC	62
1	21/2/2024	Leg Ulcers	Dr. Christopher Mwaniki-Duke	59
1	21/3/2024	Acute Chest Syndrome	Dr. Deborah Omeddo-KTRH	67
2	18/4/2024	Kidney disease in SCD	Dr. Anthony Betbadal- IHTC	54
2	23/5/2024	Splenic sequestration	Dr. Festus Njuguna- Moi Univer	95
